# Detection of carbapenemase-producing Enterobacterales by means of matrix-assisted laser desorption ionization time-of-flight mass spectrometry with ertapenem susceptibility-testing disks as source of carbapenem substrate

**DOI:** 10.3389/fmicb.2022.1059104

**Published:** 2022-11-23

**Authors:** Elvira R. Shaidullina, Andrey V. Romanov, Elena Y. Skleenova, Eugene A. Sheck, Marina V. Sukhorukova, Roman S. Kozlov, Mikhail V. Edelstein

**Affiliations:** Institute of Antimicrobial Chemotherapy, Smolensk State Medical University, Smolensk, Russia

**Keywords:** MALDI-TOF MS, resistance detection, antibiotic resistance, carbapenemases, Enterobacterales

## Abstract

MALDI-TOF mass spectrometry has become widely used in clinical microbiology and has proved highly accurate for detection of carbapenemases in Gram-negative bacteria. However, the use of carbapenem-hydrolysis assays in routine diagnostics is hampered by the need for antibiotic substances and for making their fresh solutions each time an assay is conducted. Here, we evaluated the use of commercial antibiotic susceptibility-testing disks as source of ertapenem substrate in MALDI-TOF MS-based assay for detection of carbapenemase-producing Enterobacterales (CPE). The assay was validated on 48 CPE isolates of 8 different species expressing NDM-, VIM-, KPC- and OXA-48-type carbapenemases and exhibiting various levels of resistance to carbapenems (MIC range: 0.25– > 32 mg/l), as well as on 48 carbapenemase-non-producing isolates. The assay conditions were optimized as follows: 10-μl loopful of bacterial colonies was suspended in 150 μl 0.01 M Na-PBS buffer, pH 7.4, a 10 μg ertapenem susceptibility-testing disk was immersed in the suspension and incubated 3 h at 35°C, after which supernatant was obtained by centrifugation and applied on a target plate with alpha-cyano-4-hydroxycinnamic acid matrix. Mass spectra were analyzed between 440 and 560 m/z. Carbapenemase activity was detected in all tested CPE isolates by the appearance of m/z peaks corresponding to ertapenem hydrolysis products: [M_h_ + H]^+^:494.2, [M_h_ + Na]^+^:516.2, [M_h_ + 2Na]^+^:538.2, [M_h/d_ + H]^+^:450.2, [M_h/d_ + Na]^+^:472.2, and simultaneous decrease or loss of peaks of intact antibiotic: [M + H]^+^:476.2, [M + Na]^+^:498.1, [M + 2Na]^+^:520.1. No hydrolysis peaks or loss of intact ertapenem peaks were observed for carbapenemase-negative strains. We therefore report the development of a sensitive, specific and cost-effective MALDI-TOF MS-based assay for detection of CPE, which makes use of antibiotic disks readily available in most laboratories.

## Introduction

Global spread of carbapenemase-producing *Enterobacterales* (CPE) is one of the greatest antimicrobial resistance threats to modern healthcare (Marchaim et al., 2012; Doi and Paterson, 2015; Lee et al., 2016; Potter et al., 2016; Hsu et al., 2017). Effective detection of carbapenemases is important for infection control and antibiotic treatment of CPE infections and requires fast and accurate tests. A number of such tests have been developed for use in the clinical microbiology laboratories, including molecular and immunochromatographic tests that enable rapid targeted identification of the known carbapenemases and phenotypic carbapenem hydrolysis tests that provide alternative and complementary means of detecting any carbapenem-inactivating enzymes (Nordmann et al., 2012; van der Zwaluw et al., 2015; Kieffer et al., 2019; Feng et al., 2021). Hydrolysis tests have become widely used after the introduction of the Carba NP test in 2012 (Nordmann et al., 2012). Three years later, van der Zwaluw et al. (2015) proposed an alternative simple and low-cost Carbapenem Inactivation Method (CIM) making use of common susceptibility testing disks and indicator *Escherichia coli* strain for detecting hydrolysis of meropenem, which later on received several modifications and became extremely practical for routine use (Pierce et al., 2017; Jing et al., 2018; Muntean et al., 2018).

Matrix-assisted laser desorption ionization time-of-flight mass spectrometry (MALDI-TOF MS) has revolutionized modern microbiology and has found many applications not only in species identification of bacteria and fungi but also in detecting resistance mechanisms to antimicrobials (Claydon et al., 1996; Holland et al., 1996; Oviaño and Rodríguez-Sánchez, 2021; Torres-Sangiao et al., 2021; Yoon and Jeong, 2021). Lately, MALDI-TOF MS has been successfully applied to the detection of various β-lactamases, in particular carbapenemases (Burckhardt and Zimmermann, 2011; Hrabák et al., 2011). Unlike other phenotypic assays, which rely upon the use of various indirect indicators of β-lactamase-mediated hydrolysis of β-lactams, MALDI-TOF MS allows direct visualization of mass-peaks corresponding to intact β-lactam substrates and their degradation products released upon hydrolysis and, therefore, may be considered as a reference method for detecting β-lactamase activity. However, most MALDI-TOF MS-based assays for carbapenemase detection described up to date required preparation of fresh solutions of carbapenems either from chemical substances or therapeutic formulations, which made them less suitable for routine diagnostics (Hrabák et al., 2011; Hoyos-Mallecot et al., 2014; Knox et al., 2014; Papagiannitsis et al., 2015; Ghebremedhin et al., 2016; Sakarikou et al., 2017). Recently, M. Oho et al. reported on the development of MALDI-TOF MS assay with imipenem susceptibility disk and zinc sulfate solution for detection of CPE (Oho et al., 2021).

Herein, we describe the development of a modified MALDI-TOF MS assay that makes use of commercial antibiotic susceptibility-testing disks with ertapenem for highly sensitive and specific detection of CPE.

## Materials and methods

### Bacterial isolates

A representative collection of 96 non-duplicate clinical isolates from IAC collection was used. These isolates were retrospectively collected from the national sentinel surveillance program and were previously extensively characterized for susceptibility to carbapenem antibiotics using broth microdilution method, and for carbapenemase gene content using PCR and sequencing (Kuzmenkov et al., 2021). Phenotypic expression of carbapenemases was preliminary assessed using CIM test. The isolates belonged to 11 species: *Klebsiella pneumoniae* (*n* = 65), *Escherichia coli* (*n* = 10), *Enterobacter cloacae* (*n* = 5), *Proteus mirabilis* (*n* = 5), *Serratia marcescens* (*n* = 3), *Citrobacter freundii* (*n* = 2), *Klebsiella oxytoca* (*n* = 2), *Enterobacter aerogenes* (*n* = 1), *Enterobacter asburiae* (*n* = 1), *Morganella morganii* (*n* = 1), and *Proteus vulgaris* (*n* = 1). The test collection included 48 isolates producing OXA-48-like (*n* = 30), NDM (*n* = 13), VIM (*n* = 2), KPC (*n* = 1), and combination of OXA-48-like and NDM (*n* = 2) carbapenemases, which exhibited various levels of resistance to carbapenems (MIC range: 0.25– > 32 mg/l), and 48 carbapenemase-negative isolates (Supplementary Table S1).

### Matrix-assisted laser desorption ionization time-of-flight mass spectrometry assay

Several modifications of assay conditions were tested (data not shown). These included: (i) subcultivation of test isolates on Mueller-Hinton agar with or without supplementation with 10 mM zinc sulfate; (ii) preparing suspension of test cultures in pure deionized water, 0.9% non-buffered saline solution, or sodium phosphate buffered saline (Na-PBS) with or without addition of 5% propanol-2, (iii) use of susceptibility testing disks with imipenem, 10 μg, meropenem, 10 μg, doripenem, 10 μg, or ertapenem, 10 μg (Bio-Rad Laboratories, Marnes-la-Coquette, France); (iv) use of different incubation time (from 0.5 to 4 h).

The final optimized assay conditions were as follows: Bacterial isolates were recovered from storage at −70°C in glycerol-supplemented brain-heart infusion broth and subcultured for 18 h at 35°C on Mueller–Hinton agar (BBL MH II; Becton Dickinson, Sparks, MD). A10-μl loopful of bacterial colonies was suspended in 150 μl 0.01 M Na-PBS, pH 7.4, a 10 μg ertapenem susceptibility-testing disk (Bio-Rad Laboratories) was immersed in the suspension and incubated 3 h at 35°C. The suspension was then centrifuged at 14,000 rpm for 2 min. One microliter of supernatant was applied on a steel target plate on top of the pre-dried layer of MALDI matrix (1 μl of 2.5 μg/ml alpha-cyano-4-hydroxycinnamic acid; HCCA, Bruker Daltonik, Bremen, Germany) in 50% acetonitrile, 0.1% trifluoroacetic acid dried and overlaid with the second layer of the same matrix.

Antibiotic Calibration Standard (ACS, Bruker Daltonik) was used for external instrument calibration. Mass spectra were acquired between 440–560 m/z on a Microflex LT spectrometer with flexControl software, v3.4 (Bruker Daltonik). The acquisition parameters were set as follows: ion source 1, 18.98 kV; ion source 2, 16.25 kV; lens, 0.01 kV; laser frequency, 60 Hz; digitizer trigger level, 2,500 mV; laser attenuator offset, 28%; laser attenuator range, 30%; and laser range, 15–45%. Each spot was measured using 240 laser shots in groups of 40 shots per sampling area. The MS spectra were measured automatically in at least 3 repetitions and analyzed manually by flexAnalysis software, v3.4 (Bruker Daltonik) to identify intact vs. hydrolyzed ertapenem peaks (Table 1).

**Table 1:** Expected molecular masses of ionic forms of intact ertapenem and its hydrolysis products.

Intact ertapenem		Ertapenem hydrolysis products	
MW, g/mol	475.2	[M_h_ + H]^+^	494.2
[M + H]^+^	476.2	[M_h_ + Na]^+^	516.2
[M + Na]^+^	498.1	[M_h_ + 2Na]^+^	538.2
[M + 2Na]^+^	520.1	[M_h/d_ + H]^+^	450.2
[M + 3Na]^+^	542.1	[M_h/d_ + Na]^+^	472.2

M, intact molecule; M_h_, hydrolyzed molecule; M_h/d_, hydrolyzed and decarboxylated molecule.

## Results and discussion

In the initial stage of the study, we tested different assay conditions (as briefly described in Materials and methods) and use of common susceptibility disks with ertapenem, meropenem, doripenem, and imipenem as source carbapenem substrate in MALDI-TOF MS-based hydrolysis assay. Spontaneous degradation of all three carbapenems was assessed under assay conditions at different time intervals (1 h, 2 h, 3 h, and 4 h) without bacterial culture and with carbapenemase-negative control strain of *E. coli* ATCC25922. Imipenem showed spontaneous degradation, as was revealed by appearance of detectable MS peaks of hydrolysis products after ≥3 h incubation (Supplementary materials; Figure S3A). On the other hand, meropenem, doripenem and ertapenem were notably more stable. However, the mass-peaks of intact meropenem and doripenem and their degradation products were barely detectable or not detectable.

**Figure 1:**
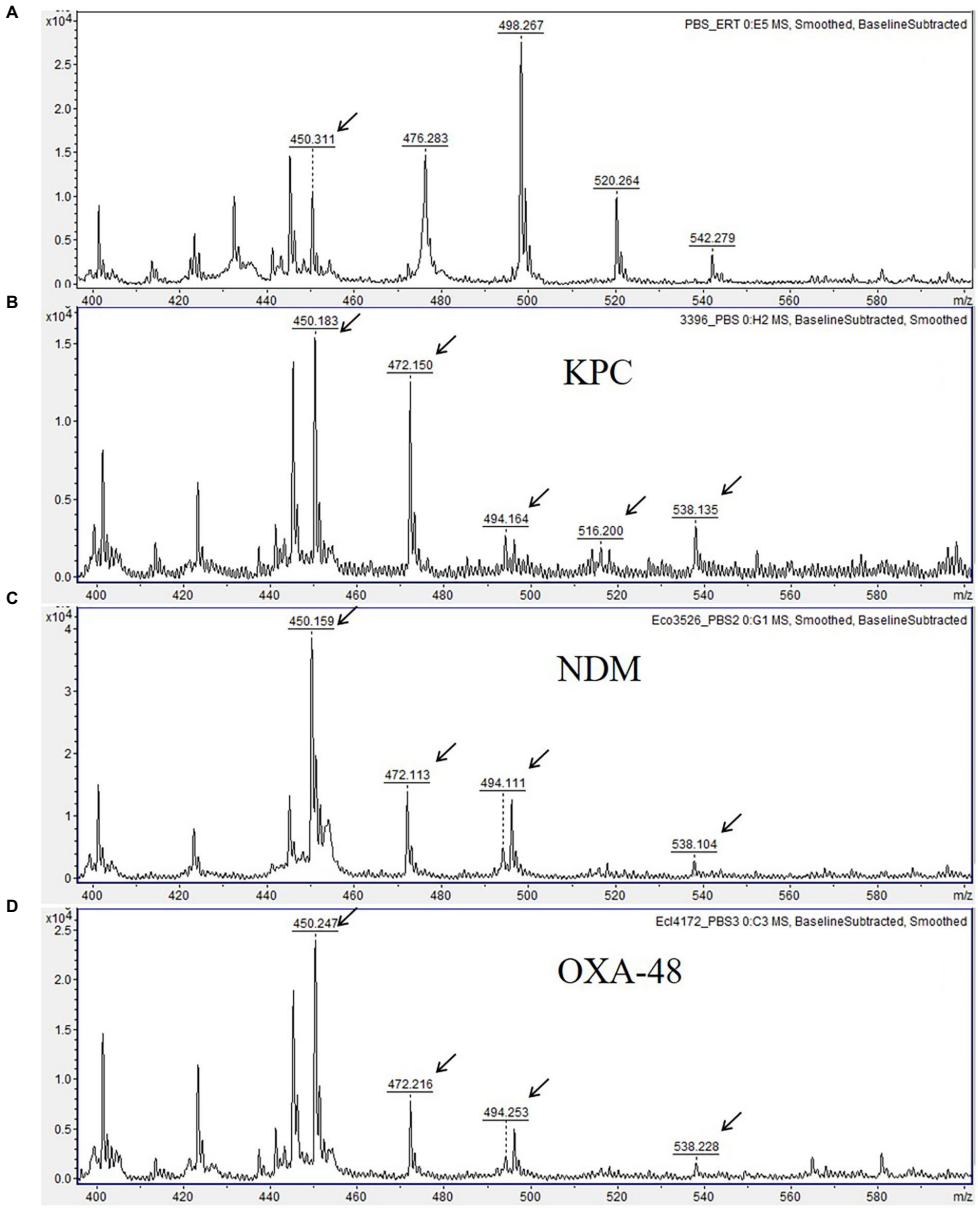
Matrix-assisted laser desorption ionization time-of-flight (MALDI-TOF) mass spectra showing ertapenem hydrolysis by carbapenemase-producing Enterobacterales. **(A)** Mass spectrum of ertapenem disk after 3 h-incubation in 0.01 M PBS, pH 7.4 (negative control). **(B–D)** Mass spectra showing ertapenem hydrolysis by KPC-3, NDM-1, and OXA-48 carbapenemases. Peaks corresponding to hydrolyzed forms of ertapenem are indicated with arrows. Units on the *Y* axes represent relative intensity.

**Figure 2:**
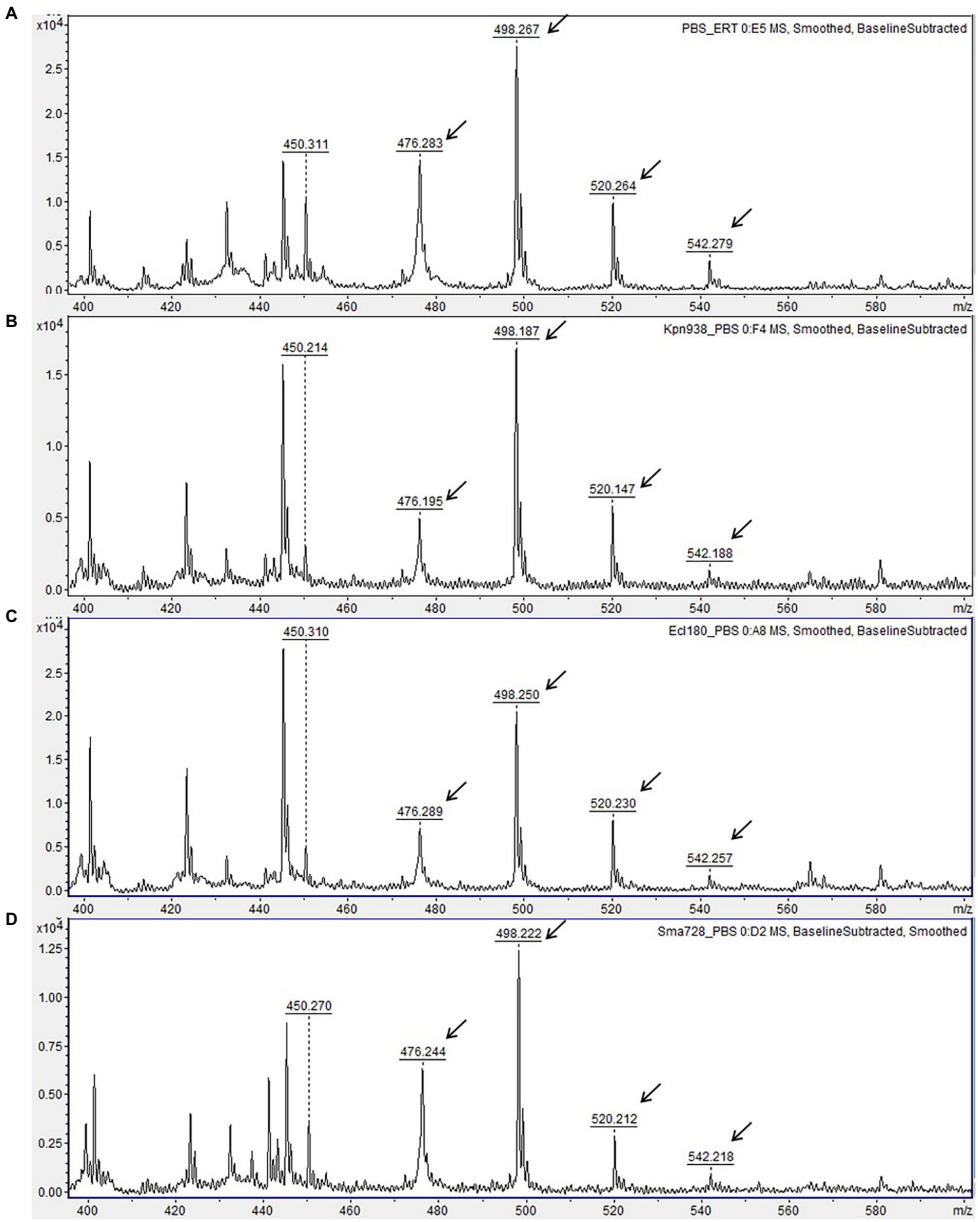
Matrix-assisted laser desorption ionization time-of-flight (MALDI-TOF) mass spectra of carbapenemase-non-producing isolates. **(A)** Mass spectrum of ertapenem disk after 3 h-incubation in 0.01 M PBS, pH 7.4 (negative control). **(B–D)** Mass spectra of ertapenem after incubation with carbapenemase-non-producing isolates of different species. Peaks corresponding to non-hydrolyzed form of ertapenem are indicated with arrows. Units on the *Y* axes represent relative intensity.

In contrast, ertapenem yielded readily visible MS peaks of intact molecule ions with high signal-to-noise ratios at a concentration of 66.6 μg/ml (0.14 mM) generated from 10-μg disk in 150 μl volume of test solution. Besides, the MS peaks of ertapenem degradation products obtained after hydrolysis were also easily detectable and distinguishable from each other and from non-specific peaks (Figures 1, 2), making ertapenem the most suitable substrate for detection of carbapenemases by means of MALDI-TOF MS. Notably, other reports (Hoyos-Mallecot et al., 2014; Sakarikou et al., 2017) have also demonstrated suitability of ertapenem as substrate for MALDI-TOF MS-based carbapenemase detection.

Using the final optimized assay conditions and incubation of bacterial cultures with ertapenem disk for up to 3 h, we detected ertapenem hydrolysis by all 48 isolates producing carbapenemases of OXA-48, KPC, NDM, and VIM groups, and exhibiting variable resistance levels to carbapenems, even with MICs below the clinical resistance breakpoints indicating weak carbapenemase expression. This was evidenced by complete or partial disappearance of MS peaks at m/z 476.2, 498.1, 520.1 and 542.1, corresponding, respectively, to intact ertapenem [M + H]^+^ and its mono- [M + Na]^+^, di- [M + 2Na]^+^, and trisodium [M + 3Na]^+^ adducts, and by simultaneous appearance of the peak at m/z 472.2 m/z corresponding to major degradation product, a monosodium adduct of hydrolyzed and decarboxylated ertapenem molecule [M_h/d_ + Na]^+^. In addition, the peaks at 494.2 m/z, 516.2 m/z and 538.2 m/z, corresponding, respectively, to the intermediate product (hydrolyzed ertapenem [M_h_ + H]^+^ and its, mono- [M_h_ + Na]^+^ and disodium [M_h_ + 2Na]^+^ ion forms) were observed for most CPE isolates (Figure 1; Supplementary Table S1).

Though a very small peak at 450.2 m/z likely corresponding to spontaneously hydrolyzed and decarboxylated ertapenem [M_h/d_ + H]^+^ was present in mass spectra of carbapenemase-negative isolates, its relative intensity was significantly smaller as compared to CPE isolates. No major hydrolysis peaks or loss of intact ertapenem peaks were observed for any of the carbapenemase-negative isolates (Figure 2). The different MS peak patterns derived from CPE and non-CPE isolates allowed their unambiguous discrimination. Therefore, the sensitivity and specificity of the developed assay reached 100% for the studied panel of isolates.

Unlike the previous study (Oho et al., 2021), we did not find supplementation of culture media with zinc sulfate to improve the detection of metallo-β-lactamases (MBLs) of NDM- and VIM-type (data not show). This may be explained by the use in our study of ertapenem instead of imipenem disks or by the cultivation of isolates on Mueller-Hinton agar which contains zinc ions in concentrations sufficient for MBLs to exert their hydrolytic activity (Asempa et al., 2021). Our study, however, did not include IMP-type MBLs that were lacking in our collection, which may be considered as a limitation of the study. Further experiments may be needed to evaluate the applicability of our assay for the detection of IMP-type carbapenemases.

## Conclusion

This study describes the development of sensitive and specific MALDI-TOF MS-based assay for detection of CPE, which makes use of materials and reagents readily available in most laboratories, such as ertapenem disks used for disk-diffusion antibiotic susceptibility testing and HCCA used as MALDI matrix for species identification of bacteria and fungi. The assay does not require highly skilled personnel, and may be used in any laboratory equipped with a MALDI-TOF mass spectrometer.

## Data availability statement

The original contributions presented in the study are included in the article/Supplementary material, further inquiries can be directed to the corresponding author.

## Author contributions

ES, ME, and RK: conceptualization. ES, AR, ElS, EuS, and MS: methodology and experimental work. ES: data validation and analysis, writing—original draft preparation. ME: writing—review and editing. RK: administration. All authors contributed to the article and approved the submitted version.

## Funding

This study was funded by Russian Federal research grant no. 1022040800475-5-1.6.2.

## Conflict of interest

The authors declare that the research was conducted in the absence of any commercial or financial relationships that could be construed as a potential conflict of interest.

## Publisher’s note

All claims expressed in this article are solely those of the authors and do not necessarily represent those of their affiliated organizations, or those of the publisher, the editors and the reviewers. Any product that may be evaluated in this article, or claim that may be made by its manufacturer, is not guaranteed or endorsed by the publisher.

## Supplementary material

SUPPLEMENTARY TABLE S1 Characteristics of the isolates used in the study and presence of the marker peaks in the corresponding mass spectra.

SUPPLEMENTARY TABLE S2 Expected molecular masses of ionic forms of intact carbapenems and their hydrolysis products.

## References

[ref1] Asempa T. E. Bajor H. Mullins J. H. Hartnett J. Nicolau D. P. (2021). Evaluation of Metallo-β-lactamase susceptibility testing in a physiologic medium. Microbiol. Spectr. 9, e01670–e01621. doi: 10.1128/Spectrum.01670-21 34817284 PMC8641967

[ref2] Burckhardt I. Zimmermann S. (2011). Using matrix-assisted laser desorption ionization-time of flight mass spectrometry to detect Carbapenem resistance within 1 to 2.5 hours. J. Clin. Microbiol. 49, 3321–3324. doi: 10.1128/jcm.00287-11 21795515 PMC3165621

[ref3] Claydon M. A. Davey S. N. Edwards-Jones V. Gordon D. B. (1996). The rapid identification of intact microorganisms using mass spectrometry. Nat. Biotechnol. 14, 1584–1586. doi: 10.1038/nbt1196-1584 9634826

[ref4] Doi Y. Paterson D. L. (2015). Carbapenemase-Producing *Enterobacteriaceae*. Semin. Respir. Crit. Care Med. 36, 74–84. doi: 10.1055/S-0035-1544208 25643272 PMC4470611

[ref5] Feng W. Niu S. Chang Y. Jia X. Huang S. Yang P. (2021). Design of Rapid Detection System for five major Carbapenemase families (bla_KPC_, *bla*_NDM_, *bla*_VIM_, *bla*_IMP_ and *bla*_OXA-48-Like_) by colorimetric loop-mediated isothermal amplification. Infect. Drug Resist. 14, 1865–1874. doi: 10.2147/IDR.S301757 34079297 PMC8164214

[ref6] Ghebremedhin B. Halstenbach A. Smiljanic M. Kaase M. Ahmad-Nejad P. (2016). MALDI-TOF MS based carbapenemase detection from culture isolates and from positive blood culture vials. Ann. Clin. Microbiol. Antimicrob. 15:5. doi: 10.1186/s12941-016-0120-x 26839024 PMC4736273

[ref7] Holland R. D. Wilkes J. G. Rafii F. Sutherland J. B. Persons C. C. Voorhees K. J.. (1996). Rapid identification of intact whole bacteria based on spectral patterns using matrix-assisted laser desorption/ionization with time-of-flight mass spectrometry. Rapid Commun. Mass Spectrom. 10, 1227–1232. 8759332 10.1002/(SICI)1097-0231(19960731)10:10<1227::AID-RCM659>3.0.CO;2-6

[ref8] Hoyos-Mallecot Y. Cabrera-Alvargonzalez J. J. Miranda-Casas C. Rojo-Martín M. D. Liebana-Martos C. Navarro-Marí J. M. (2014). MALDI-TOF MS, a useful instrument for differentiating metallo-β-lactamases in *Enterobacteriaceae* and *pseudomonas* spp. Lett. Appl. Microbiol. 58, 325–329. doi: 10.1111/lam.12203 24286119

[ref9] Hrabák J. Walková R. Študentová V. Chudáčková E. Bergerová T. (2011). Carbapenemase activity detection by matrix-assisted laser desorption ionization-time of flight mass spectrometry. J. Clin. Microbiol. 49, 3222–3227. doi: 10.1128/JCM.00984-11 21775535 PMC3165603

[ref10] Hsu L. Y. Apisarnthanarak A. Khan E. Suwantarat N. Ghafur A. Tambyah P. (2017). Carbapenem-resistant *Acinetobacter baumannii* and *Enterobacteriaceae* in south and Southeast Asia. Clin. Microbiol. Rev. 30, 1–22. doi: 10.1128/CMR.00042-16 27795305 PMC5217790

[ref11] Jing X. Zhou H. Min X. Zhang X. Yang Q. Du S.. (2018). The simplified Carbapenem inactivation method (sCIM) for simple and accurate detection of Carbapenemase-producing gram-negative bacilli. Front. Microbiol. 9:2391. doi: 10.3389/FMICB.2018.02391 30425686 PMC6218411

[ref12] Kieffer N. Poirel L. Nordmann P. (2019). Rapid immunochromatography-based detection of carbapenemase producers. Infection 47, 673–675. doi: 10.1007/S15010-019-01326-1 31144273

[ref13] Knox J. Jadhav S. Sevior D. Agyekum A. Whipp M. Waring L.. (2014). Phenotypic detection of carbapenemase-producing *Enterobacteriaceae* by use of matrix-assisted laser desorption ionization-time of flight mass spectrometry and the Carba NP test. J. Clin. Microbiol. 52, 4075–4077. doi: 10.1128/JCM.02121-14 25187633 PMC4313257

[ref14] Kuzmenkov A. Y. Trushin I. V. Vinogradova A. G. Avramenko A. A. Sukhorukova M. V. Malhotra-Kumar S.. (2021). AMRmap: an interactive web platform for analysis of antimicrobial resistance surveillance data in Russia. Front. Microbiol. 12:377. doi: 10.3389/FMICB.2021.620002 PMC7994358 33776956

[ref15] Lee C. R. Lee J. H. Park K. S. Kim Y. B. Jeong B. C. Lee S. H. (2016). Global dissemination of carbapenemase-producing *Klebsiella pneumoniae*: epidemiology, genetic context, treatment options, and detection methods. Front. Microbiol. 7:895. doi: 10.3389/FMICB.2016.00895 27379038 PMC4904035

[ref16] Marchaim D. Chopra T. Bhargava A. Bogan C. Dhar S. Hayakawa K.. (2012). Recent exposure to antimicrobials and Carbapenem-resistant *Enterobacteriaceae*: the role of antimicrobial stewardship. Infect. Control Hosp. Epidemiol. 33, 817–830. doi: 10.1086/666642 22759550 PMC4370272

[ref17] Muntean M.-M. Muntean A.-A. Gauthier L. Creton E. Cotellon G. Popa M. I.. (2018). Evaluation of the rapid carbapenem inactivation method (rCIM): a phenotypic screening test for carbapenemase-producing *Enterobacteriaceae*. J. Antimicrob. Chemother. 73, 900–908. doi: 10.1093/jac/dkx519 29351668

[ref18] Nordmann P. Poirel L. Dortet L. (2012). Rapid detection of Carbapenemase-producing *Enterobacteriaceae*. Emerg. Infect. Dis. 18, 1503–1507. doi: 10.3201/eid1809.120355 22932472 PMC3437707

[ref19] Oho M. Funashima Y. Nagasawa Z. Miyamoto H. Sueoka E. (2021). Rapid detection method of carbapenemase-producing Enterobacteriaceae by MALDI-TOF MS with imipenem/cilastatin (KB) disc and zinc sulfate solution. J. Infect. Chemother. 27, 205–210. doi: 10.1016/J.JIAC.2020.09.013 33008738

[ref20] Oviaño M. Rodríguez-Sánchez B. (2021). MALDI-TOF mass spectrometry in the 21st century clinical microbiology laboratory. Enferm. Infecc. Microbiol. Clin. 39, 192–200. doi: 10.1016/J.EIMC.2020.02.027 32345492

[ref21] Papagiannitsis C. C. Študentová V. Izdebski R. Oikonomou O. Pfeifer Y. Petinaki E.. (2015). Matrix-assisted laser desorption ionization-time of flight mass spectrometry Meropenem hydrolysis assay with NH_4_HCO_3_, a reliable tool for direct detection of Carbapenemase activity. J. Clin. Microbiol. 53, 1731–1735. doi: 10.1128/JCM.03094-14 25694522 PMC4400744

[ref22] Pierce V. M. Simner P. J. Lonsway D. R. Roe-Carpenter D. E. Johnson J. K. Brasso W. B.. (2017). Modified Carbapenem inactivation method for phenotypic detection of Carbapenemase production among *Enterobacteriaceae*. J. Clin. Microbiol. 55, 2321–2333. doi: 10.1128/JCM.00193-17 28381609 PMC5527410

[ref23] Potter R. F. D’Souza A. W. Dantas G. (2016). The rapid spread of carbapenem-resistant *Enterobacteriaceae*. Drug Resist. Updat. 29, 30–46. doi: 10.1016/j.drup.2016.09.002 27912842 PMC5140036

[ref24] Sakarikou C. Ciotti M. Dolfa C. Angeletti S. Favalli C. (2017). Rapid detection of carbapenemase-producing *Klebsiella pneumoniae* strains derived from blood cultures by matrix-assisted laser desorption ionization-time of flight mass spectrometry (MALDI-TOF MS). BMC Microbiol. 17:54. doi: 10.1186/S12866-017-0952-3 28274205 PMC5343375

[ref25] Torres-Sangiao E. Leal Rodriguez C. García-Riestra C. (2021). Application and perspectives of MALDI–TOF mass spectrometry in clinical microbiology laboratories. Microorganisms 9:1539. doi: 10.3390/microorganisms9071539 34361974 PMC8307939

[ref26] van der Zwaluw K. De Haan A. Pluister G. N. Bootsma H. J. De Neeling A. J. Schouls L. M. (2015). The Carbapenem inactivation method (CIM), a simple and low-cost alternative for the Carba NP test to assess phenotypic Carbapenemase activity in gram-negative rods. PLoS One 10:e0123690. doi: 10.1371/journal.pone.0123690 25798828 PMC4370852

[ref27] Yoon E.-J. Jeong S. H. (2021). MALDI-TOF mass spectrometry technology as a tool for the rapid diagnosis of antimicrobial resistance in bacteria. Antibiotics 10:982. doi: 10.3390/antibiotics10080982 34439032 PMC8388893

