# Supplementary material for: Detection of carbapenemase-producing Enterobacterales by means of matrix-assisted laser desorption ionization time-of-flight mass spectrometry with ertapenem susceptibility-testing disks as source of carbapenem substrate
Source: Front Microbiol. 2022 Nov 23;13:1059104. doi: 10.3389/fmicb.2022.1059104 (PMC9727098; doi:10.3389/fmicb.2022.1059104)
Supplement: SUPPLEMENTARY TABLE S2 — Expected molecular masses of ionic forms of intact carbapenems and their hydrolysis products. [file Data_Sheet_1.docx]

**Table S2.** Expected molecular masses of ionic forms of intact carbapenems and their hydrolysis products.

|  | Ertapenem | Imipenem | Meropenem | Doripenem |
| --- | --- | --- | --- | --- |
| MW, g/mol | 475.2 | 299.3 | 383.4 | 420.5 |
| Intact forms | | | | |
| [M+H]^+^ | 476.2 | 300.3 | 384.4 | 421.5 |
| [M+Na]^+^ | 498.1 | 322.3 | 406.4 | 443.5 |
| [M+2Na] ^+^ | 520.1 | 344.3 | 428.4 | 465.5 |
| [M+3Na] ^+^ | 542.1 |  |  |  |
| Hydrolysis products | | | | |
| [M_h_+H] ^+^ | 494.2 | 318.3 | 402.4 | 439.5 |
| [M_h_+Na] ^+^ | 516.2 | 340.3 | 424.4 | 461.5 |
| [M_h_+2Na] ^+^ | 538.2 | 362.3 | 446.4 | 481.5 |
| [M_h/d_+H] ^+^ | 450.2 | 274.3 | 358.4 | 395.5 |
| [M_h/d_+Na] ^+^ | 472.2 | 296.3 | 380.4 | 417.5 |

M, intact molecule; M_h_, hydrolyzed molecule; M_h/d_, hydrolyzed and decarboxylated molecule.

The corresponding mass-spectra are shown in **Figure S3** in the next page.

**
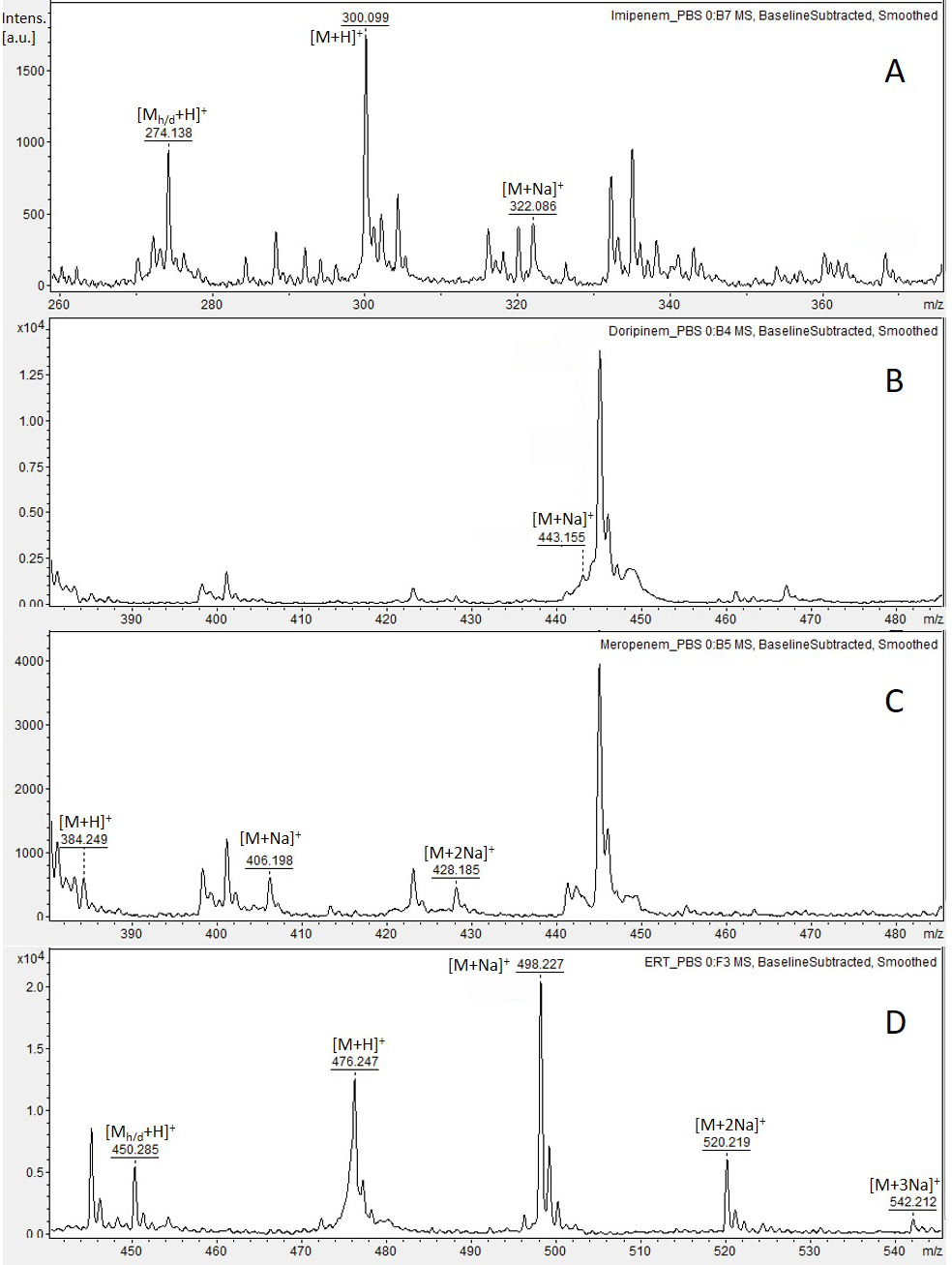
**

**Figure S3.** MALDI-TOF mass spectra of four carbapenem disks after 3h-incubation in 0.01M PBS.

A, Imipenem. (Note a relatively high peak of spontaneously hydrolyzed and decarboxylated imipenem [M_h/d_+H]^+^ at 274.2 m/z); B, Doripenem. (Note a small peak of monosodium adduct at 443.2 m/z. Other ionic forms are not detectable); С, Meropenem. (Note three peaks of intact meropenem and its mono- and disodium adducts at 384.2, 406.2 and 428.8 m/z, which have at least 30-times lower intensities than ertapenem peaks); D, Ertapenem (Note clearly detectable peaks of intact ertapenem adducts at 476.3, 498.2, 520.2 and 542.2 m/z and relatively small peak of hydrolyzed and decarboxylated ertapenem at 450.3 m/z).
